# Participation of Latin American surgeons in Twitter using the hashtag #SoMe4Surgery and #SoMe4IQLatAm

**DOI:** 10.1016/j.sopen.2022.03.008

**Published:** 2022-03-31

**Authors:** Laura B. Castro, Luis F. Cabrera, Mariana Reyes, Mauricio Pedraza, Ivan David Lozada-Martinez, Nicolas Forero, Sabrina Rahman

**Affiliations:** a Semillero de Investigación en Cirugía General y Subespecialidades Department of General Surgery, Universidad El Bosque, Bogotá, Colombia; b Vascular Surgery and Angiology Department, Hospital Militar Central, Bogotá, Colombia; c Medical and Surgical Research Center, Future Surgeons Chapter, Colombian Surgery Association, Bogotá, Colombia; d Grupo Prometheus y Biomedicina Aplicada a las Ciencias Clínicas, School of Medicine, Universidad de Cartagena, Cartagena, Colombia; e School of Medicine, Universidad de los Andes, Bogotá, Colombia; f Independent University, Dhaka, Bangladesh

## Abstract

**Background:**

In medicine, social networks contribute to the professional training because it is a way to improve the knowledge and skills of students, residents and specialists; additionally, these networks allow the dissemination of evidence. However, Latin American surgeons' influence within this social network is not highlighted. In this study, using the hashtags #SoMe4Surgery and #SoMe4IQLatAm, the participation of Latin American surgeons in Twitter is established.

**Study Design:**

This is a prospective cross-sectional study of the academic tweets published in the period between October 13 and October 19, 2020, on Twitter by the academic accounts @Cirbosque and @MISIRG1 who are users of the social network Twitter in Latin America who participate in the network with the hashtags #SoMe4Surgery and #SoMe4IQLatAm with academic use.

**Results:**

A total of 56 tweets and 665 retweets were analyzed. Male sex presents greater interaction, as well as Mondays and Tuesdays week days. Geolocation was recorded as 37.1% in Latin America and 17.6% in Europe. #SoMe4Surgery was mentioned in 31 tweets and generated 211,700 impressions and 25,557 interactions, and #SoMe4IQLatam was mentioned in 25 tweets and achieved 57,585 impressions and 21,901 interactions. A growth of the participation rate of 6.5% in @Cirbosque and 10.5% in @ MISIRG1 was estimated for 2021.

**Conclusion:**

The use of social networks, particularly Twitter, in the surgeon community has proven to be a valuable tool during the last decade. The tweet that needs to be shared among more surgeons should be linked to the hashtag #SoMe4IQLatAm and #SoMe4Surgery and Twitter surgeon leaders mentions.

## INTRODUCTION

Over time, technology and digital tools have become an indispensable part of the academic and professional development [1]. In medicine, social networks contribute to the professional training because it is a way to improve the knowledge and skills of students, residents, and specialists; additionally, these networks allow the dissemination of studies, publications, conferences, and clinical cases and interaction with other colleagues [[2], [3], [4], [5], [6], [7]]. Particularly, among the contributions of social networks to surgical medical education are mentoring, globalization, generation of a digital educational platform, virtual simulation of procedures, research and collaboration, information access, and training opportunities [8,9].

All this evolution of surgery social networks has generated an impact that has gradually increased the number of surgeon users on Twitter and has enabled the formation of new groups and hashtags. An example is the hashtag #SoMe4Surgery, created on July 28, 2018, to promote inclusive and multidisciplinary surgery, bringing together doctors from all over the world and catalyzing surgical research activity on social media. The hashtag #SoMe4IQLatAm was created on January 2019 with the purpose of joining together all the surgical opinions of the Latin American surgeons in the social media Twitter, but its use is not widespread and until now it is underused [8].

The analysis of the interaction on Twitter began in North America and Europe, and the impact on the social network of the accounts of the main surgical journals and surgical mentors from those countries is currently being defined. However, the Latin American surgeons' influence within this social network is not highlighted. It is important to establish the grade of use of social media for surgical academic purposes by the Latin American surgeons to define the utility of this applications to networking, research, education, promotion, and leadership in the Latin American surgery and its dissemination around the world. In this study, using the hashtags #SoMe4Surgery and #SoMe4IQLatAm from 2 Colombian accounts, @Cirbosque and @MISIRG1, 2 of the most influential and engaging accounts around the surgical world in the social media Twitter with more than 10,000 followers and more than 11,000,000 of interactions, we evaluate the academic participation of Latin American surgeons in the social network Twitter.

## MATERIALS AND METHODS

A prospective cross-sectional study of the academic tweets published in the period between October 13 and October 19, 2020, on Twitter by the academic accounts @Cirbosque and @MISIRG1 (4 tweets each day in each account at the following times: the first at 7:00, the second at 10:00, the third at 12:00, and the last at 18:00; each tweet had the hashtag #SoMe4IQLatAm or #SoMe4Surgery) and the students, interns, general surgery residents, and specialists who are users of the social network Twitter in Latin America who participate in the network with the hashtags #SoMe4Surgery and #SoMe4IQLatAm with academic use. We analyzed only the specific tweets performed using the hashtags #SoMe4Surgery and #SoMe4IQLatAm in the 2 accounts during the study period. The data analysis was performed with the help of a specialist in social media data analysis and community manager.

**Fig 1-2:**
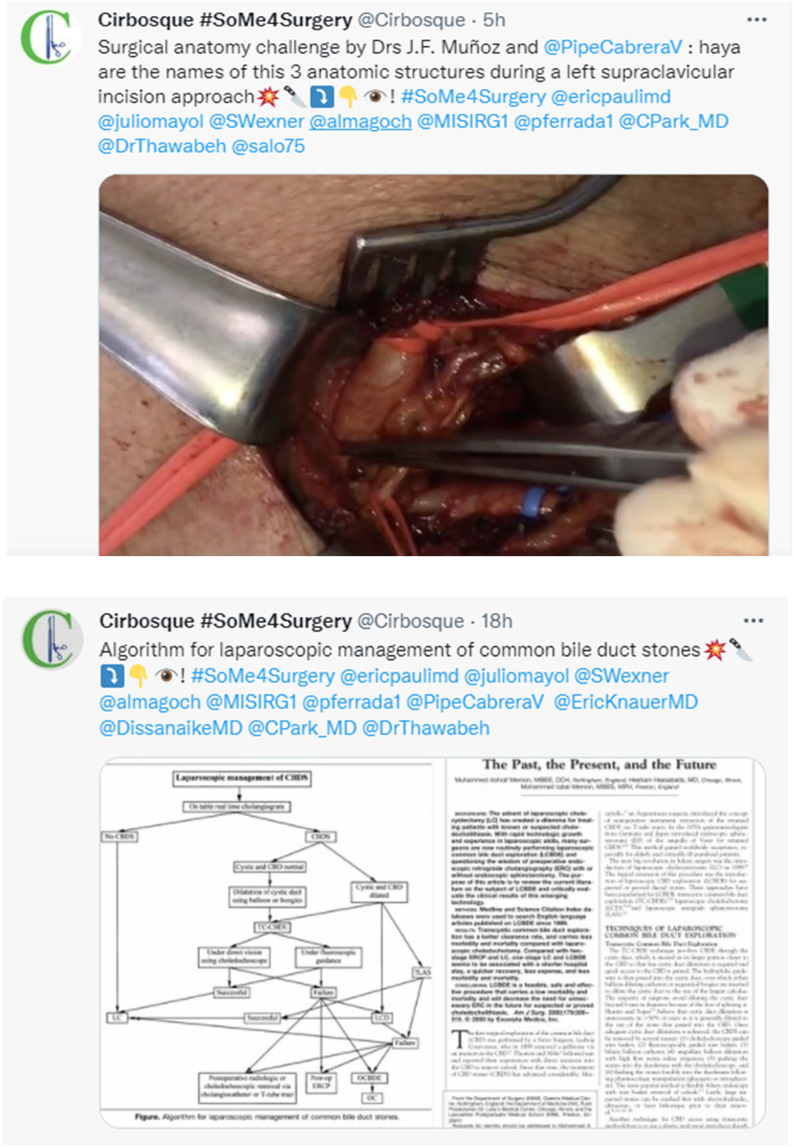
Example of tweets from the academic surgery accounts studied.

The @cirbosque and @MISIRG1 are 2 academic twitter accounts created in December 2018 to promote the surgical significant learning using social media of the general surgery residency program of Universidad El Bosque. All the tweets performed in this 2 accounts contain a short academic title, the academic hashtag, and the most influential surgeons on Twitter plus a video or an image related to the short title to improve the academic surgical information. In these 2 accounts are performed a lot of types of academic surgical tweets, like image challenges, video challenges, surgical case discussions, academic tweets, surgeons in history, and arts in surgery (Figs 1–2).

To obtain these data, Twitter Analytics established the variables to get at Twitter Application Programming Interface (API) where an API Key and an APISecret were generated to access a temporary connection key. The procedure was programmed in Data Science language (R or Python), and a script was configured to extract the data to be analyzed. With the get-statuses-lookup method, the initial metadata of the tweets were obtained; with the get_retweets method, the base of retweets was obtained; and with the get_retweeters method, the data of the users who retweeted were obtained. This process was carried out on November 27, 2020, the cutoff date established for data collection.

**Fig 3:**
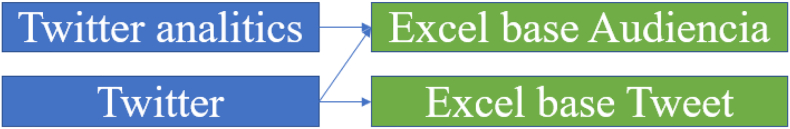
Data source diagram**.** Source: authors.

To identify the sex and location of the users who retweet and do not identify themselves in the tool, the accounts of each user were reviewed, identifying the words and images of their profile to determine the sex and location. In Figure 3, the flow diagram of the information obtained in the data collection is observed. (See Fig 1-2.)

For the statistical analysis of the results, the Office Excel program was used. The qualitative variables were presented in terms of proportions, frequencies, and graphs. The analysis of the information obtained was carried out with comparative analysis of the engagement variables, impressions, retweets, likes, tweets with the hashtags # SoMe4IQLatAm or # SoMe4Surgery, the classification of the content of each tweet, and the location and sex of the users.

### Definitions

Hashtag: A topic identifier in a tweet or other social network message that is indexed by the service so that all the user postings with that hashtag can be searched and read. The hashtag is the name of the topic with a number sign (#) prefix, also called a hash mark. Hashtags are created for anything and everything, including people, businesses, organizations, sports teams, political parties, hobbies, events and philosophies, as well as rants and raves.

Engagement: The number of interactions your content received from users (likes, comments, shares, saves, etc).

Impressions: The number of times your content is displayed.

Reach: The number of people who see your content.

Engagement Rate: Impressions Engagement Rate informs about the percentage of users who engaged with a post during its lifetime given its impressions. The Impressions Engagement Rate metric shows organic and boosted data (note that Instagram metrics gather organic data only).

Interactions: The effectiveness of your social media campaigns at fostering positive engagement. Interaction: A communication between an audience member and your brand's social profile [[1], [2], [3]].

### Ethical and Legal Aspects

This research did not require the approval of the ethics committee or the elaboration of an informed consent. It complies with Resolution 008430 of 1993 of the Ministry of Health of Colombia. No conflict of interest or source of financing is declared. All the authors declare to have contributed to the entire conception and design of the study, acquisition, analysis and interpretation of data, and writing and critical review of the manuscript.

## RESULTS

A total of 56 tweets were published by the accounts @Cirbosque (10,100 followers) and @MISIRG1 (6,567 followers); each account published 28 tweets, 4 per day. The data analyzed show the hashtags #SoMe4IQLatAm and #SoMe4Surgery interactions; the number of tweets, engagement, impressions, and retweets (Table 1); and according to the content classification, the engagement, impressions, retweets, and likes (Table 2).

**Table 1:** Comparative table of each hashtag interactions

*Hashtag*	*Tweets*	*Engagement*	*Impressions*	*Retweets*
SoMe4Surgery	31	25,557	211,700	345
SoMe4IQLatam	25	21,901	157,785	320
Total	56	Not apply	Not apply	665

**Table 2:** Classification of academic tweets according to their content, engagement, impressions, retweets, and generated likes

*Classification/Subclassification*	*Engagement*	*Impressions*	*Retweets*	*Likes*
1	14.870	75.696	95	430
2	5.331	24.386	18	94
3.1	4.334	51.942	77	231
3.2	5.581	51.271	124	460
3.3	14.825	119.516	270	952
3.4	1.555	31.035	54	125
4	106	2.341	2	6
5	856	13.298	25	60

The activity of each account, @Cirbosque and @MISIRG1, was determined by engagement and impressions. The @Cirbosque account obtained 24,431 engagements with 7,041 and 170,276 impressions. The @MISIRG1 account obtained 23,027 engagements and 199,209 impressions.

The days and time of greatest interaction of the 2 accounts, @Cirbosque and @MISIRG1, are Monday with the greatest engagement and impressions and Tuesday with the greatest retweets and likes generated (Table 3, Table 4).

**Table 3:** Days of the week classified by engagement, impressions, retweets, and likes

*Week day*	*Engagement*	*Impressions*	*Retweets*	*Likes*
Monday	10.580	65.004	88	317
Tuesday	5.626	57.993	147	458
Wednesday	6.746	53.138	115	393
Thursday	9.588	56.077	102	430
Friday	7.369	53.372	89	341
Saturday	4.031	40.550	75	249
Sunday	3.518	43.351	49	170

**Table 4:** Time of publication of the tweet in relation with engagement, impressions, retweets, and likes

*Time*	*Engagement*	*Impressions*	*Retweets*	*Likes*
0700	7.188	81.133	154	541
1000	15.985	104.054	186	690
1200	17.048	116.015	170	657
1800	7.237	68.283	155	470

A total of 665 retweets were generated, 278 made by @Cirbosque account and 387 by @MISIRG1 account; retweets generated by each tweet were organized from the highest to the lowest number of retweets per account to establish the first 5 tweets. The most common sex of the users who interacted with @Cirbosque and @MISIRG1 accounts is men (74.4%). The geolocation participation showed 37.1% in Latin America followed by Europe with 17.6% and then Asia, North America, Africa, and Oceania. However, 16% do not report georeferencing; subsequently, the country of the Latin American users was identified as Colombia being the first one (46.2%) followed by Mexico (26.3%) and Venezuela (6.5%).

The engagement rate for the year 2019 compared to the same dates in 2020 as well as a prospective calculation for the year 2021 was generated to determine how strategies and planning of academic tweet publications in general surgery with clinical cases and academic topics are required to achieve impact, loyalty, influence, and recognition. The @Cirbosque engagement rate for 2019 is 1.2% compared to 4.2% in 2020; for @MISIRG, it was 11.6% and 4.1%, respectively. It is estimated that the engagement rate will have a growth of 6.5% in @Cirbosque and 10.5% in @MISIRG1 by 2021.

According to the use of the hashtag #SoMe4Surgery between October 13 and 19, 2020, it was mentioned in 31 tweets, which generated 345 retweets, achieving a total of 211,700 impressions and generating 25,557 interactions between users. Unlike the previous one, for the hashtag #SoMe4IQLatam, during the same period of time, it was mentioned in 25 tweets, generating 320 retweets and achieving a total of 157,785 impressions, which in turn generated 21,901 interactions.

## DISCUSSION

With the incursion of advanced technology, virtual spaces such as social networks have become the protagonists in the management of massive information. The possibility of communicating without barriers has generated the use of these by specialists from all professions, in which medicine has not been the exception. In addition, easy access at a low cost and the possibility of using different multimedia formats for information management increase their use. For this reason, they are very useful for surgery, facilitating dialogue between surgeons from anywhere in the world, who share experiences and analyze surgical techniques through images and videos. Not knowing the possibilities of these digital tools for general surgery represents losing possibilities of learning, contacts, and professional visibility.

The practice of *medicine* is defined as the "set of knowledge and techniques applied to the prediction, prevention, diagnosis and treatment of human diseases and, where appropriate, to the rehabilitation of the consequences that may occur" [3]. Surgeons must have an intensive academic preparation; years of study and practice; and interaction with teachers, patients, colleagues, professional associations, and networks. Books, academic journals, and other publications are essential as well [10,11]. Faced with this need, the use of social networks has shown possibilities to strengthen training and surgical medical practice, allowing contact with people from all over the world, globalizing access to information, and demolishing economic and geographic barriers [1].

Surgeons and general surgery residents have benefited from access to multimedia resources, which was previously zero, limited, or outdated. Online videos are now used for surgical learning, in which various "studies have shown the benefits of multimedia in the learning process, specifically in the conversion of cognitive information into long-term memory, indicative of learning" [12]. In the light of its utility, The Andalusian Society of Surgeons publishes on its portal each year the number of users on the Internet and social networks [13].

Among the countless social platforms, Twitter is undoubtedly the one that has positioned itself worldwide as the most powerful channel for managing collective information. This network is characterized by its immediacy, brevity, and universality, with the option of including links, photographs, and videos, as well as generating conversations [14,15]. The Twitter potential lies in the professional opportunity to build academic communities and research networks, as well as to present findings, achievements, and concerns in their action fields [16]. According to the Andalusian Association of Surgeons, this microblogging network is the one that has the most acceptance and use among surgeons to exchange all kinds of content publicly, and depending on the Surgeon users' participation, it will be possible to achieve greater scientific global dissemination, strengthening knowledge and achieving a positive impact on clinical practice of students, teachers, and graduates of general surgery. It also makes possible to publish institutional content, making the practice and academic achievements visible and putting the name of universities and their community on the world stage [8,13].

To understand the incursion in social networks of general surgeons in Latin America being a great dissemination, acquisition, and updating tool of knowledge, as well as a new form of interaction between surgeons [17], the publication of scheduled tweets during the week between October 13 and 19, 2020, of the academic accounts @Cirbosque and @MISIRG1 was monitored and analyzed; also the hashtags #SoMe4Surgery and #SoMe4IQLatAm have a common objective through their use, sharing and invigorating information that increases the connection between users and creates academic discussion opportunities.

#SoMe4Surgery mentions, retweets, impressions, and interactions between October 13 and 19, 2020, and #SoMe4IQLatam data during the same period show that linking tweets with a hashtag like #SoMe4Surgery results in a greater number of views and interaction between the audience, given that it belongs to a global ecosystem unlike the hashtag #SoMe4IQLatAm, which has a smaller and more selected ecosystem limited to Latin America. The #SoMe4Surgery hashtag has greater engagement and generates more views.

The highest visibility impressions and engagements in both accounts were obtained among tweets with content classification number 1 and 2, presentation of surgical academic cases, and identification of diagnosis with clinical image. That's how tweets that include clinical cases and clinical images in their content achieve a greater impact given the number of interactions and activity, reaching a greater diffusion among users, which is in accordance with international literature. In the case of surgery, the content in video format, mostly dedicated to the detailed description of surgical techniques, has increased in recent years [18]. On the other hand, tweets that make presentation and dissemination of an international publication of a surgical academic issue are in second place according to the interactions generated among users, especially when referring to surgical techniques, which caused greater interest, followed by topics such as surgical pathologies, surgical anatomy, and finally current issues. In the analysis, regarding the number of impressions, @MISIRG1 account had a better performance during the analyzed period. However, for the number of impressions achieved by the tweets, the @Cirbosque account achieved the highest engagement among the audience.

On the other hand, the days with the greatest interaction and interest for the users of the @Cirbosque and @MISIRG1 accounts are from Monday to Thursday because they are weekdays when the majority of surgeons are on duty; during this time, they can publish and discuss clinical cases and surgical topics of interest. Between Friday and Saturday, there is a decrease in activity, and finally on Sunday, there are no interactions between users. The time where the greatest interaction of the users of the @Cirbosque and @ MISIRG1 accounts is recorded is between 1200 and 1300 probably because it is the rest space after the morning shift of rounds of medical consultations, surgeries, and consultations. Associated with this, 0700 is where there is the least interaction and activity probably because this is the time when everyone comes to the hospital and examines patients.

The @Cirbosque registers a higher number of followers (10,100) than @MISIRG1 (6,567). However, @Cirbosque presents a total 170,276 impressions compared to the 199,209 impressions of the @MISIRG1 account, which indicates that this figure is not directly proportional to the number of followers.

Retweets from each account present content on the most common procedures performed by general surgeons, such as the correction of inguinal hernias and the abdominal wall closure technique. For example, tweet number 4 of @MISIRG1 achieved the highest number of retweets, doubling the number of retweets of @Cirbosque tweet number 6. Its content corresponds to the 10 commandments that surgeons must take into account to perform abdominal wall closure after any intra-abdominal procedure, topics that surgeons should always be clear about for all intra-abdominal procedures that are performed, being common in emergencies and in ambulatory surgery.

In addition, the participation of men was higher in both accounts, which coincides with the statistics of the social network Twitter users and the statistics in the world where there is a higher prevalence of male surgeons over female surgeons associated with the gradual insertion that women in surgery have had since the 20th century, so strategies should be designed for greater women inclusion and participation on these settings. A high number of users do not register personal data such as age and sex in their biography on Twitter, which do not allow an analysis of the information and modify the results; for example, in the Latin American audience, we have interaction with 243 retweets, but only 2 accounts have personal data in their biography such as age.

The geolocation participation showed 37.1% in Latin America followed by Europe (17.6%). Latin America has a greater participation given that the accounts are academic surgical institutional accounts of Colombia and Latin America with an audience composed mainly of undergraduate and graduate students and professors with publications focused on surgical interest directly related to their population. In Latin America, different surgical influencers are documented, such as, in Mexico, Dr Alberto Gonzales Chavez, a general surgeon at the Hospital Español de México, with his account @almagoch with 26,100 followers since 2010 and Dr Mario Gonzales with his account @MarioGonzlez with 9,365 followers since 2010 and, in Central America, Dr Danilo Acevedo in Nicaragua. It is also highlighted that a large part of the audience are residents of general surgery in other countries such as Venezuela and Mexico.

In Latin America, the 5 countries with the highest participation are Colombia as first with a 46.2% participation, with Bogotá and Medellín being the cities with the highest activity, followed by Mexico, the second country with the largest population and more activity in the @MISIRG1 account. Venezuela is the third country in Latin America with the highest activity, which is explained because most accounts' activity in the experiment correspond to surgical residents, who, given the current crisis in the country, are required to be updated with activities, procedures, and technologies that are being used in the nearest countries.

Europe is one of the main continents that have ventured with the presence of surgeons in social networks, mainly in Spain, where, since 2013, the participation analysis of the general surgeons in the annual congresses and meetings of the associations has been carried out since 2013. In this country, we find one of the surgeons who started and who has been an influencer of the presence of surgeons in social networks, Dr Julio Mayol, General Surgeon of the Clinical Hospital in Madrid, with his account @juliomayol, which has around 45,000 followers since 2009.

Likewise, there was great participation from Asia, mainly in the countries of the Middle East and India with the account @MISIRG1 in front of @Cirbosque because its approach is based on research topics on minimally invasive laparoscopic surgery and discussion topics on the evolution and novel procedures in surgery [19].

North America, given that it is one of the pioneers in the presence of surgeons on Twitter, has a greater presence, having as references the accounts of groups such as JAMA Surgery (@JAMASurgery), which began tweeting in 2009; Annals of Surgery (@AnnalsofSurgery) in 2011; Journal of the American College of Surgeons (JACS) (@JAmCollSurg) in 2013; the New England Journal of Medicine (@NEJM); and the American Medical Association (@AmerMedicalAssn). It presents a greater participation in the @MISIRG1 account because the base of the account is in minimally invasive research where it is one of the countries with the most technology.

The participation rate of the institutional academic accounts @Cirbosque and @MISIRG1 during 2019 was lower than 2%, showing little interaction with followers. However, for the same period of 2020, the percentage was greater than 4%, which improved with the management of the information to be published and by maintaining an active and constant participation that increases the interaction and community interest, reaching 499,500 impressions by @Cirbosque and 301,000 impressions corresponding to @MISIRG1; also, they reported the highest number of published tweets, reaching up to 221 in @Cirbosque and 90 tweets in @ MISIRG1, achieving better performance with the design of strategies, starting with the hashtag use and regarding the importance of ethical and professional use. Following this statement, a growth of 6.5% in the engagement rate was obtained; however, growth among time is expected to be higher.

In conclusion, the use of social networks, particularly Twitter, in the surgeon community has proven to be a valuable tool during the last decade. The connection between surgeons on Twitter offers opportunities to discuss research and clinical practice issues at an international level, and linking tweets with a hashtag generates more visits and interactions. The tweet that needs to be shared among more surgeons should be linked to the hashtag #SoMe4IQLatAm and #SoMe4Surgery and Twitter surgeon leaders mentions. Publications may be about clinical cases, clinical images, surgical pathologies, and surgical technique anatomy, which have proven to be effective for the creation of a network of professionals linked to the field of surgery, encouraging interest in the construction and dissemination of knowledge and the generation of a playful and pedagogical educational scenario based on collaboration and use of virtual tools.

## Conflict of Interest

The authors have no conflicts of interest to disclose.

## Funding Source

This research did not receive any specific grant from funding agencies in the public, commercial, or not-for-profit sectors.

## Ethics Approval

This study was approved by the ethics committee of our hospital.
